# Total and Regional White Matter Lesions Are Correlated With Motor and Cognitive Impairments in Carriers of the *FMR1* Premutation

**DOI:** 10.3389/fneur.2019.00832

**Published:** 2019-08-13

**Authors:** Darren R. Hocking, Danuta Z. Loesch, Nicholas Trost, Minh Q. Bui, Eleanor Hammersley, David Francis, Flora Tassone, Elsdon Storey

**Affiliations:** ^1^Developmental Neuromotor and Cognition Lab, School of Psychology and Public Health, La Trobe University, Melbourne, VIC, Australia; ^2^School of Psychology and Public Health, La Trobe University, Melbourne, VIC, Australia; ^3^Department of Radiology, St. Vincent's Hospital Melbourne, Fitzroy, VIC, Australia; ^4^Centre for Molecular, Environmental, Genetic and Analytic Epidemiology, University of Melbourne, Melbourne, VIC, Australia; ^5^VCGS Cytogenetics Laboratory, Murdoch Children's Research Institute, Royal Children's Hospital, Melbourne, VIC, Australia; ^6^Department of Biochemistry and Molecular Medicine, University of California, Davis, Davis, CA, United States; ^7^School of Medicine, MIND Institute, University of California Davis Medical Center, Davis, CA, United States; ^8^Department of Medicine (Neuroscience), Central Clinical School, Monash University, Melbourne, VIC, Australia

**Keywords:** *FMR1* premutation carriers, FXTAS, MRI, white matter hyperintensities, motor scores, cognitive impairment

## Abstract

This study explores the relationships between hemispheric and cerebellar white matter lesions and motor and cognitive impairments in male carriers of Fragile-X Mental Retardation 1 (*FMR1*) premutation alleles, and in a subgroup of these carriers affected with Fragile X-Associated Tremor/Ataxia syndrome (FXTAS). Regional and total white matter hyperintensities (*wmhs*) on MRI, assessed using semiquantitative scores, were correlated with three motor rating scales (ICARS, UPDRS, Tremor), and neuropsychological measures of non-verbal reasoning, working memory and processing speed, in a sample of 30 male premutation carriers aged 39–81 years, and separately in a subsample of 17 of these carriers affected with FXTAS. There were significant relationships between *wmhs* in the infratentorial region and all three motor scales, as well as several cognitive measures—Prorated IQ, Matrix Reasoning, Similarities, and the Symbol Digit Modalities Test (SDMT), in the total sample of carriers, as well as in the FXTAS group separately. This shows that *whms* within the infratentorial region correlates across the categories of clinical status with a range of motor and cognitive impairments. In the FXTAS group, there was a highly significant relationship between supratentorial (periventricular) lesions and parkinsonism, and between both periventricular and supratentorial deep white matter and ICARS ataxia score. These findings further support the relevance of white matter changes in different brain regions to the motor and cognitive deficits across the spectrum of premutation involvement. Future longitudinal studies using larger sample sizes will be necessary to examine the factors that lead to conversion to a greater extent of neurological involvement as seen in the progression across the FXTAS spectrum.

## Introduction

The most severe form of clinical disorder associated with premutation expansions (55–200 CGG repeats) in the 5′ untranslated region of the Fragile X Mental Retardation 1 (*FMR1*) gene is a late-onset progressive neurodegenerative condition: Fragile X-Associated Tremor/Ataxia Syndrome (FXTAS) (1). This syndrome usually occurs after, and progresses from, the age of 55 years, and is more prevalent in male (45%) than in female (8–16%) carriers of the premutation allele. The clinical features include intention and/or postural tremor, cerebellar ataxia, dementia, and parkinsonism in some cases. Major neuroradiological features include white matter hyperintensities (*wmhs*) in the middle cerebellar peduncles (“MCP sign”) and/or in the splenium of the corpus callosum (2). Occasionally, a milder version of this syndrome has been reported in carriers of “gray zone” *FMR1* alleles with CGG expansions ranging between 41 and 54 CGGs (3–5).

The mechanisms involved in neural pathology associated with premutation alleles (PM) are not fully understood. However, evidence suggests several possible pathomechanisms: a “toxic” gain of function of the elevated and expanded *FMR1* mRNA resulting in sequestration of proteins needed for normal neuronal functioning (6); the cellular aggregation of “toxic” peptides as a result of repeat-associated non-ATG (RAN) translation of the CGG repeat; and cellular dysregulation involved in the DNA damage response to hairpin R-loops formed by *FMR1* mRNA expanded CGG repeats (7).

Further neuroradiological and neuropathological studies in FXTAS patients have provided important data concerning the neuropathological underpinnings of the spectrum of premutation involvement, with evidence suggesting preferential white matter lesions within cerebral and cerebellar hemispheres on magnetic resonance imaging (MRI), associated with general cerebral and cerebellar atrophy and enlargement of the lateral ventricles (8, 9). Neuropathological studies have also revealed cerebral and cerebellar white matter degeneration, with widespread loss of myelin and axons alongside preserved frontal cortical thickness and neuronal density (10). More recent studies have shown a significant prevalence of *wmhs* in the splenium of the corpus callosum (2, 11), which is now one of the major radiological diagnostic features, or in the basis points (12) in FXTAS patients, in addition to the most characteristic MCP sign. Although gray matter volume loss has also been demonstrated in patients with FXTAS in both cortical and subcortical regions (13, 14), the observation of white matter abnormalities in PM carriers with and without FXTAS (8, 15) suggests that white matter pathology is the *primum movens*. Moreover, the cognitive profile of executive dysfunction, slowed processing speed, deficits in working memory and declarative memory alongside preserved language, supports the notion that FXTAS is primarily a white matter disease (16). Diffusion tensor imaging (DTI) studies have shown loss of white matter integrity extending beyond the cerebellar peduncles and the corpus callosum (14, 17, 18). These white matter abnormalities affect the structural connectivity of numerous fiber tracts relevant to motor control, as well as specific cognitive abilities. Notably, changes in diffuse white matter within cerebral hemispheric and cerebellar regions, as well as volume changes in the cerebellum, brain stem, and whole brain, have been reported in PM carriers with and without FXTAS (15, 17). This suggests that white matter alterations may serve as sensitive markers of incipient decline to FXTAS, or to asyndromic neurological manifestations not meeting the diagnostic criteria for FXTAS. This aspect was discussed in our earlier publication (19), where we reported an example of the presence of the MCP sign in some non-FXTAS PM carriers, consistent with similar observations in five such individuals most recently reported by Famula et al. (20).

The existing evidence from neuroradiological and clinical observations, combined with the recent report of the presence of intranuclear inclusions in both FXTAS and non-FXTAS (including asymptomatic) PM carriers (21), further suggests the continuity of underlying structural pathology within the CNS associated with the *FMR1* premutation carriage. This white matter pathology may correspond to the severity and type of neurological manifestations, with the most severe form represented by clinical and structural brain changes as seen in FXTAS.

The current study aimed to examine the relationships between severity of white matter lesions and motor and cognitive impairments in older males carrying the PM allele with and without FXTAS. All three motor scores, assessing ataxia, tremor and parkinsonism (see methods for details), and a limited range of cognitive impairments, were shown to be associated with the extent of white matter lesions in the infratentorial region in the total sample of males with the PM. This suggests that the varying degree and extent of neuropathological processes, unites all carriers of the premutation allele, ranging from asymptomatic to non-syndromic features to the full manifestation of FXTAS.

## Methods and Materials

### Participants

The study was approved by the La Trobe University Human Ethics Committee (No. 01/85). All participants gave both written and informed consent for their involvement. The sample comprised 30 older men with small CGG repeat expansions: 29 with the *FMR1* PM, and one borderline PM/gray-zone carrier with 54 repeats (referred to collectively as “PM carriers”). Seventeen of these 30 participants had a diagnosis of FXTAS, of whom one participant was classified as FXTAS solely on the basis of the MCP sign associated with dementia. Amongst 13 carriers without FXTAS, seven participants manifested other disorders or non-syndromic changes outside the FXTAS spectrum, including: fibromyalgia (1); isolated dementia (2); isolated intention tremors that were associated with minor *wmh* changes but no MCP sign (3); and orthostatic tremor (4). Six carriers were not affected. Altogether, we included 17 FXTAS and 13 non-FXTAS participants totalling 30 PM carriers.

The sample was drawn from two studies occurring between the years 2004–2008 (5 carriers with FXTAS), and in the years 2012–2016 inclusive (25 carriers, 12 with FXTAS and 13 non-FXTAS). The ages ranged from 50 to 81 years (M = 63.0) for the FXTAS group, and from 55 to 69 years (M = 57.7 years), with one outstanding younger individual aged 39, for PM carriers without FXTAS. CGG repeat sizes in the PM carriers ranged from 56 to 160, and the participant with a PM/gray zone borderline value had 54 repeats. The *FMR1* mRNA levels ranged from 2.32 to 5.38 units, compared with the baseline (control) value of 1.0 (6). All participants were recruited from families that were identified through clinical diagnoses of children with Fragile X syndrome (FXS: > 200 CGG repeats) as determined by the Victorian Clinical Genetic Service at the Royal Children's Hospital in Melbourne.

### Neuroimaging

Brain MRI scans were acquired using a 1.5 Tesla Siemens scanner located at St. Vincent's Hospital Melbourne. Scans were captured using turbo spin-echo 2 dimensional (i) proton-density with T2 weighting (TR 3500, TE 13/103) and/or (ii) fluid-attenuated inversion recovery (FLAIR) (TR 9000, TE 90, TI 2500) axial images with a 5 mm slice thickness or 3D FLAIR (TR 5800, TE 315, TI 2200) with 1 mm voxel size.

#### Visual White Matter Hyperintensity Rating

The extent and severity of *wmhs* in the infratentorial region (infra-DWMH), and the deep and the periventricular supratentorial regions (Supra-DWMH and PV-WMH, respectively) were evaluated from the proton-density/T2 and/or the FLAIR images by one experienced neuroradiologist (NT) using a visual semi-quantitative method. The evaluation was performed blinded to clinical data and repeat size, and was repeated in a subsample of 10 participants after 6 months by the same neuroradiologist, with a consistency approaching 100%. The DWMH rating was based on the method described by Wahlund et al. (22). Since DWMH and PV-WMH are likely to result from different pathological processes and vary in extent and severity between different clinical scenarios (23) and a review of previous studies (24, 25) demonstrated increased PV-WMH in FXTAS subjects compared with normal controls, they were separately rated as described by other authors (23, 26).

DWMHs were defined as areas of increased T2 signal > 3 mm in diameter, and were rated, in four supratentorial regions, for each side of the brain: frontal; parieto-occipital; deep temporal; and subcortical white matter. In infratentorial regions, DWMHs included the MCP T2 hyperintensities that constitute one of the major criteria for the diagnosis of FXTAS, as well as T2 hyperintensities in adjacent deep white matter of the cerebellar hemispheres. The basal ganglia (BG) regions, which encompassed the deep nuclei and internal and external capsules, were not included in the ratings.

**Figure 1:**
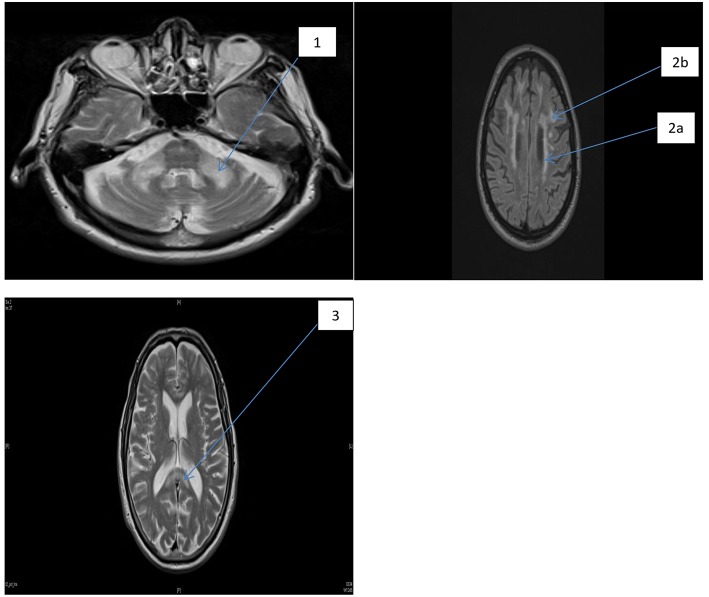
FLAIR MRI axial section of two representative patients with FXTAS. MCP sign and foci of *wmhs* (1), the periventricular *wmhs* (2a), deep *wmhs* (2b), and *wmhs* in the splenium of the corpus callosum (3).

PV-WMHs were defined as confluent hyperintensities adjacent to the frontal or occipital horns (caps) or the bodies (bands) of the lateral ventricles. When PV-WMH extended >10 mm from the ventricular surface they were given a score of two, and any excess was included in the DWMH score. Respective scores for individual regions listed above were totalled to give: total supratentorial DWMH (“Total Supra-DWMH”), total infratentorial DWMH (“Total Infra-DWMH”), and total periventricular WMH (“Total PV-WMH”) scores, and the sum of these three regional summary scores was labeled as “Total-WMH”. Chronic lacunes, which were identified as well-defined areas >3 mm with signal characteristics similar to cerebrospinal fluid, were rare and were not included in either the *wmhs* rating or in other analyses. A detailed description of the measures used, together with illustration of the spectrum of changes, has been provided in a previous study (27). Figure 1 shows the *wmhs* in the supratentorial (left) and infratentorial (right) regions as well as changes in the corpus callosum in two representative patients with FXTAS.

### Neuropsychological and Motor Rating Assessments

A battery of tests were selected to evaluate cognitive status with special emphasis on working memory and processing speed, which are domains predominantly affected in FXTAS (16, 28). General cognitive ability was assessed by the Vocabulary and Matrix Reasoning subtests of the Wechsler Adult Intelligence Scale (Third Edition; WAIS-III), which were used to calculate a prorated Full Scale IQ score (29). Verbal and non-verbal reasoning were evaluated using the Similarities and Matrix Reasoning subtests of the WAIS-III. The Digit Span forward and backward scores from the WAIS-III Digit Span subtest were selected as measures of short-term verbal memory and working memory, respectively. Information processing speed was assessed with the Symbol Digit Modalities Test (SDMT) (30). Severity of motor dysfunction was evaluated using the International Cooperative Ataxia Rating Scale (ICARS) (31), the Unified Parkinson's Disease Rating Scale (UPDRS) Part III-Motor (32), and the Clinical Rating Scale for Tremor (33). The assessments and scoring were conducted by two experienced neurologists (ES & DZL).

### Molecular Analyses

CGG repeat expansion size was derived from previous diagnostic testing of fragile X families at the Victorian Clinical Genetic Services, Melbourne. PCRs and Southern Blot analyses were used and all assays were fully validated by internal and external quality assessment to provide a precision of ± one repeat (34, 35). Total RNA was isolated from 3 mL of blood collected in Tempus tubes (Applied Biosystems, Foster City, California, USA) or from 1 × 10^6^ cells using Trizol (Life Technologies, Carlsbad, California, USA). The measurement of *FMR1* mRNA expression levels was carried out by quantitative Real Time qRT-PCR on total RNA using custom-designed Taqman gene expression assays (Applied Biosystems) as previously described (36).

### Statistical Analysis

Descriptive statistics were presented as means and standard deviations for the total sample of premutation carriers, and separately for FXTAS and Non-FXTAS groups. The relationship between each neuropsychological or motor score, and each *wmh s*core, was assessed using the least square regression method if outliers were not present, otherwise robust regression was used to downweight the influential effect of outliers. Age was included as a covariate in the final regression for all the measures. The results of regression analysis are presented for the FXTAS and Non-FXTAS samples combined if there was no significant group interaction, with 2-sided *p*-value of <0.1 considered significant. The interaction term was incorporated in the multiple linear regression model, where each motor score or cognitive score were outcome variables, and predictors were individual *wmhs* scores, FXTAS group (binary), age (if significant) and interaction between FXTAS group and *wmhs* scores. The significance of the interaction term implies that estimated regression coefficients are different between the two groups, and the results presented for FXTAS group alone have been based on analysis using parameters extracted from the interaction model. Significance of regression was assessed by using the one-tailed test in cases where the direction of the relationship was predictable, with the *p*-value <0.0125 considered significant after adjustment for multiple testing (of the four *wmh* scores) conducted using the Bonferroni correction method. All analyses were carried out using STATA statistical software (version 13, STATA Corporation, 2013).

## Results

Table 1 shows the descriptive statistics on age, CGG repeat number and the levels of *FMR1* mRNA for FXTAS and Non-FXTAS groups separately and combined.

**Table 1:** Descriptive statistics for sample characteristics for the total sample of PM carriers and separately for FXTAS and non-FXTAS groups.

	**Combined**			**FXTAS**			**Non-FXTAS**		
	**Mean**	**SD**	**Range**	**Mean**	**SD**	**Range**	**Mean**	**SD**	**Range**
**CHARACTERISTIC**									
Age	60.6	10.2	39–81	63.3	8.31	50–81	57.1	11.6	33.0–69
CGG	77.2	19.4	51–118	89.1	16.5	62–118	62.5	10.8	51.0–84
FMR1 mRNA	2.94	1.35	1.4–6.0	2.89	1.20	1.4–5.4	3.12	1.98	1.50–6.0

Tables 2A,B presents the results of regression analyses showing relationships between *wmhs* scores and motor or neuropsychological scores, with motor or cognitive score as outcome measures, and *wmhs s*cores as predictors. The results for motor scores (in Table 2A) are shown for the FXTAS group alone, and for the total sample of premutation carriers only in cases where the interaction term from regression analysis was not significant.

**Table 2A:** Relationships between each motor score (outcome) and each *wmhs* score (predictor), adjusted for age, and the FXTAS group.

	**All**						**FXTAS**				
	***N***	**Coef**	**s.e**	***F*_**(df1, df2)**_**	***p***	***R*^**2**^**	**Coef**	**s.e**	***F*_**(df1, df2)**_**	***p***	***R*^**2**^**
**TOTAL WMH**											
UPDRS	28						0.244	0.101	5.86 (1, 23)	**0.012**	0.45
ICARS	28						0.803	0.170	22.3 (1, 23)	**4.0 × 10**^**−5**^	0.71
Tremor scale	22	0.617	0.255	5.86 (1, 19)	0.013	0.24	0.277	0.319	0.76 (1, 17)	0.199	
**SUPRA- DWMH**											
UPDRS	28	0.297	0.274	1.17 (1, 25)	0.145	0.13	0.443	0.293	2.28 (1, 23)	0.072	
ICARS	28						2.475	0.424	34.1 (1, 23)	**3.0 × 10**^**−6**^	0.75
Tremor scale	22	0.789	0.771	1.04 (1, 19)	0.160	0.05	0.289	0.794	0.13 (1, 17)	0.360	
**INFRA- DWMH**											
UPDRS	28	0.642	0.193	11.0 (1, 25)	**0.002**	0.41	0.451	0.270	2.79 (1, 23)	0.055	
ICARS	28	2.547	0.474	28.9 (1, 25)	**7.0 × 10**^**−6**^	0.57	2.048	0.694	8.70 (1, 23)	**0.004**	
Tremor scale	22	1.827	0.367	24.8 (1, 19)	**4.2 × 10**^**−6**^	0.58	6.617	3.239	4.16 (1, 17)	0.040	
**PV-WMH**											
UPDRS	28						0.806	0.223	13.1 (1, 23)	**0.0005**	0.60
ICARS	28	1.093	0.582	3.53 (1, 25)	0.036	0.15	1.477	0.513	8.29 (1, 23)	**0.005**	
Tremor scale	22	2.146	2.096	1.04 (1, 19)	0.160	0.10	1.440	2.301	0.40 (1, 23)	0.270	

*Results for the total sample of PM carriers (FXTAS and Non-FXTAS groups combined) were shown only in cases where interaction was not significant. N, sample size; Coef, estimated regression coefficient; s.e, standard errors; F, F- statistics; df_1_, numerator's degrees of freedom; df_2_, denominator's degree of freedom; P-value (p) in each FXTAS group was 1-sided; p values in bold indicate that a relationship remains significant (p < 0.05) after adjustment for multiple testing*.

**Table 2B:** Relationship between each cognitive score (outcome) and each *wmhs* score (predictor), adjusted for age, in the total sample of PM carriers.

	***N***	**Coef**	**s.e**	***F*_**(df1, df2)**_**	***p***	***R*^**2**^**
**TOTAL WMH**						
Prorated IQ	30	−0.704	0.322	4.75 (1, 27)	0.019	0.15
Similarity	30	−0.094	0.080	1.39 (1, 27)	0.125	0.06
Vocabulary	26	−0.110	0.069	2.59 (1, 23)	0.061	0.10
Matrix reasoning	26	−0.145	0.068	4.58 (1, 23)	0.022	0.17
Digit span	26	−0.145	0.056	6.66 (1, 23)	**0.009**	0.23
SDMT RS	15	−0.667	0.332	4.04 (1, 12)	0.034	0.46
**SUPRA- DWMH**						
Prorated IQ	30	−1.012	0.855	1.39 (1, 27)	0.124	0.05
Similarity	30	−0.032	0.200	0.03 (1, 27)	0.436	0.01
Vocabulary	26	−0.164	0.176	0.86 (1, 23)	0.181	0.04
Matrix reasoning	26	−0.128	0.181	0.50 (1, 23)	0.243	0.02
Digit span	26	−0.256	0.156	2.72 (1, 23)	0.057	0.11
SDMT RS	15	−0.973	0.908	1.14 (1, 12)	0.153	0.34
**INFRA- DWMH**						
Prorated IQ	30	−1.879	0.720	6.81 (1, 27)	**0.008**	0.20
Similarity	30	−0.440	0.168	6.81 (1, 27)	**0.007**	0.21
Vocabulary	26	−0.350	0.155	5.11 (1, 23)	0.017	0.18
Matrix reasoning	26	−0.474	0.160	8.76 (1, 23)	**0.004**	0.28
Digit span	26	−0.268	0.145	3.42 (1, 23)	0.039	0.14
SDMT RS	15	−2.667	0.670	15.8 (1, 12)	**0.001**	0.67
**PV -WMH**						
Prorated IQ	30	−1.600	1.029	2.43 (1, 27)	0.066	0.09
Similarity	30	−0.035	0.245	0.02 (1, 27)	0.444	0.01
Vocabulary	26	−0.164	0.217	0.58 (1, 23)	0.228	0.03
Matrix reasoning	26	−0.401	0.226	3.13 (1, 23)	0.045	0.12
Digit span	26	−0.332	0.185	3.24 (1, 23)	0.043	0.14
SDMT RS	15	−1.116	1.234	0.81 (1, 12)	0.192	0.25

*N, sample size; Coef, estimated regression coefficient; s.e, standard errors; F, F- statistics; df_1_, numerator's degree of freedom; df_2_, denominator's degrees of freedom; p, 1-sided p-value; R^2^, R-square. P-values in bold indicate that a relationship remains significant (p < 0.05) after adjustment for multiple testing*.

In the FXTAS group, the ICARS (ataxia score)—the measure most closely reflecting the neurological spectrum of FXTAS- was significantly associated with *wmhs* scores from all supratentorial, as well as infratentorial regions. The UPDRS showed a significant association with the Total WMH score, but the association was exceptionally high with the periventricular *wmhs* (PV-WMH), with the *p* value reaching 0.0005, compared with *p* = 0.012 for the total WMH, which includes all the regions.

The results from the total sample show highly significant associations between all three motor scale scores (including Tremor score) and Infra-DWMH representing the infratentorial locations of *wmhs* (see Table 2A). The relationships between the two supratentorial *wmhs* (Supra-DWMH and PV-WMH) with ICARS and Tremor scale, and between Supra-DWMH and UPDRS were not significant in the total sample.

The results of regression for the cognitive test scores are presented for the FXTAS and Non-FXTAS groups combined (Table 2B) considering that the interaction term did not reach significance for all the relationships involving these scores (*p* values ranging from 0.119 to 0.943).

**Figure 2:**
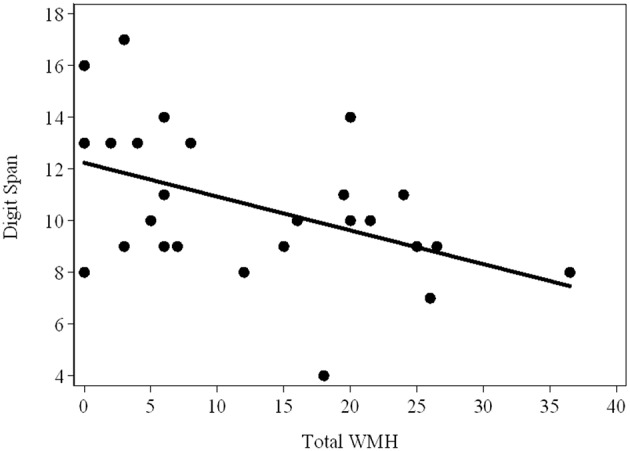
Scatterplot showing the (raw) Total Digit Span scores against the WMH scores in the total sample (FXTAS and Non FXTAS combined) of PM carriers.

As shown in Table 2B, there were strong relationships of Prorated IQ (*p* = 0.008), SDMT (*p* = 0.001), Matrix Reasoning (*p* = 0.004), and Similarity (*p* = 0.007) with Infra-DWMH, but all remaining correlations did not survive adjustment for multiple testing. There was also a highly significant (negative) correlation between Digit Span and Total WMH (*p* = 0.009) (Figure 2). The scatterplot of this correlation illustrates a continuity of the relationships across the total sample of PM carriers, ranging from apparently asymptomatic throughout asyndromic to affected FXTAS carriers.

## Discussion

This is the first study to explore relationships of white matter lesions (*whms*) in several defined areas of the brain with the wide range of clinical measures including motor features of tremor, ataxia and parkinsonism, and neuropsychological measures of working memory and processing speed in older male carriers of the *FMR1* premutation. We have shown that *whms* within the infratentorial region is associated with impairments across the categories of clinical status for all the three motor rating scores, and a range of neuropsychological cognitive measures. This is supportive of the notion that the scope of the spectrum of premutation-related clinical and brain changes, especially those in infratentorial regions specifically affected in FXTAS, appear to extend beyond the classical syndromic forms of FXTAS, and across the categories of non-affected and non-syndromic carriers, comprising asymptomatic, asyndromic and FXTAS groups (19). In addition, in the FXTAS group we have demonstrated a highly significant relationship between supratentorial (periventricular) lesions and parkinsonism, and between both periventricular and supratentorial deep white matter lesions and the ICARS ataxia score. Furthermore, our data demonstrate that white matter lesions in infratentorial regions—the regional *wmhs* most characteristic of FXTAS—are significantly (negatively) correlated with a range of cognitive scores, namely processing speed, matrix reasoning and similarities, as well as Prorated IQ, in the total sample of carriers. These findings further support the relevance of white matter changes in different brain regions to the motor and cognitive deficits across the spectrum of premutation involvement.

Notably, when taken together with our previous study (19), the current findings imply a continuity in the clinical and white matter changes extending from non-affected older male carriers, to non-syndromic forms separate from FXTAS, and eventually to the full manifestation of FXTAS. Moreover, the finding of significant correlations between the *wmhs* in infratentorial regions and the motor and cognitive scores in the total sample of PM carriers suggests that the underlying primary pathology might be initially confined to this region. These findings corroborate previous studies using more sophisticated techniques to examine white matter microstructure *in vivo* by the sensitive MRI technique of DTI (15, 17). These authors speculated that the presymptomatic alterations in structural connectivity seen in several brain regions in non-affected premutation carriers may represent a subgroup at risk of developing clinical manifestations of FXTAS with increasing age. Moreover, the significant associations between reduced tract volume in the superior cerebellar peduncle and CGG repeat size in both unaffected premutation carriers and those with FXTAS (37) provides support for a continuum of white matter changes irrespective of the clinical category. It is therefore possible that this underlying pathology, if aggravated by aging and other possible genetic or non-genetic risk factors including stress-related cellular damage, could progress and expand beyond the “primary” infratentorial area to involve the supratentorial regions leading to the more severe clinical changes seen in FXTAS.

Although our data on the relationships between white matter lesions and cognitive scores were less clear, the significant correlations which survived correction for multiple comparisons in the combined PM sample: between infratentorial *wmhs* score and each of the non-verbal problem solving and processing speed scores, constitute major aspects of cognitive functioning known to be impaired in FXTAS (16). Our findings are generally concordant with the results of the first study of the relationship between white matter disease and cognitive functioning in a small sample of 13 older male PM carriers (8). In contrast, this study applied more advanced and sensitive MRI techniques than the conventional MRI imaging employed in the current study—DTI and magnetic resonance spectroscopy (MRS)—and showed that microstructural white matter abnormalities in the MCPs and the genu and splenium of the corpus callosus, were correlated with executive dysfunction and slowed processing speed (8). The most interesting result from that study revealed decreased levels of N-acetyl aspartate (NAA) in the MCP, which suggested the presence of axonal damage (8). Although direct comparison between findings from that study and our own results is not possible, given the differences in MRI techniques employed (*wmh* rating scale vs. DTI and MRS), and the utilization of different cognitive measures (Matrix Reasoning vs. Behavioral Dyscontrol Scale and COWAT), both studies' results suggest that the dominant lesion in the white matter of the cerebellar peduncles in PM carriers might disrupt fronto-cerebellar networks, which in turn could affect input from the contralateral cerebral cortex to the inferolateral cerebellum, thus leading to the non-motor impairments seen in affected PM carriers (38). Notably, both these studies have been consistent in showing the continuity of neural pathology from non-FXTAS to the full manifestation of FXTAS.

There are several limitations of our study that merit consideration. The small sample size combined with the inclusion of multiple measures in regression analysis may have accounted for a limited number of significant correlations between cognitive functioning and the hemispheric white matter lesions, and also for a number of significant interactions for FXTAS group that might be due to small sample size and considerable variability. Another limitation is the lack of a control group with normal *FMR1* alleles with comparable age range to evaluate severity of pathological processes specifically related to the continuity of the changes in the *FMR1* premutation. Future studies using longitudinal designs, with larger cohorts of PM carriers including those affected with FXTAS, asymptomatic carriers, those affected with non-syndromic neurological features and controls are necessary to confirm the postulated early clinical and neurodegenerative changes, which may lead to divergent clinical outcomes. Using conventional MRI with semiquantitative lesion scores may also be considered a limitation, since it does not permit more sensitive and tract-based analysis of white matter microstructure. However, this approach clearly served the purpose of our study, which aimed to establish whether white matter lesions in supratentorial, as well as infratentorial regions, contribute to motor and cognitive changes characteristic of the spectrum of premutation involvement. The validity of the *wmh* scores applied in this study was further attested by demonstration of the highly significant associations of all the *wmhs* scores used here with the levels of “toxic” *FMR1* mRNA in a previous study (19). Finally, our study design did not include examination of the gray matter changes that have been reported by others in FXTAS (13, 14, 39, 40).

In summary, our results provide preliminary evidence for the relevance of the regional and total macrostructural white matter lesions to the neurological changes occurring in *FMR1* premutation carriers, by finding linear relationships between infratentorial *wmhs* and measures of motor and specific cognitive impairments across all categories of clinical status. Further studies based on larger cohorts of premutation carriers and longitudinal modeling are needed to confirm our hypothesis that the neural and clinical changes may occur across the entire spectrum of PM carriers with continuity of the underlying neurodegenerative process. Notably, our results have suggested an involvement of the locations other than infratentorial region in this process; however, the specific factors that lead to progressive changes that involve the deep hemispheric and periventricular regions in the severity of motor impairments in the full manifestation of FXTAS remains to be determined. By using sensitive MRI techniques such as DTI and MRS, these studies will enable assessment of microstructural white matter changes and gray matter loss, and their association with motor and cognitive impairments reminiscent of FXTAS. This may reveal the earliest changes that precede the severe neurodegeneration associated with obvious clinical manifestations seen in FXTAS, and thereby lead to identification of targets for therapeutic intervention.

## Data Availability

The datasets generated from the statistical analysis for this study are available on request to the corresponding author.

## Ethics Statement

This study was approved by the La Trobe University Human Ethics Committee (No. 01/85). All participants gave informed consent for their involvement.

## Author Contributions

DH wrote the first draft of the manuscript and provided intellectual input into the interpretation of the data. DL conceptualized and designed the study, provided intellectual input into the interpretation of the data, and co-wrote the first draft of the manuscript. NT contributed to MRI protocol and ratings and input on the interpretation of the findings. MB provided statistical analysis and input into interpretation of the data. EH conducted neuropsychological testing. DF and FT conducted genetic molecular *FMR1* testings. EH, DF, and FT provided intellectual input into interpretation of data. ES assessed the subjects neurologically and contributed to drafting the final version of the manuscript.

### Conflict of Interest Statement

The authors declare that the research was conducted in the absence of any commercial or financial relationships that could be construed as a potential conflict of interest.
